# Lightsheet optical tweezer (LOT) for optical manipulation of microscopic particles and live cells

**DOI:** 10.1038/s41598-022-13095-3

**Published:** 2022-06-17

**Authors:** Partha Pratim Mondal, Neptune Baro, Ankur Singh, Prakash Joshi, Jigmi Basumatary

**Affiliations:** 1 Department of Instrumentation and Applied Physics, Indian Institute of Science, Bangalore, 560012 India; 2 Applied Photonics Initiative, Indian Institute of Science, Bangalore, 560012 India

**Keywords:** Biological techniques, Biotechnology, Cell biology, Medical research

## Abstract

Optical trapping and patterning cells or microscopic particles is fascinating. We developed a light sheet-based optical tweezer to trap dielectric particles and live HeLa cells. The technique requires the generation of a tightly focussed diffraction-limited light-sheet realized by a combination of cylindrical lens and high NA objective lens. The resultant field is a focussed line (along *x*-axis) perpendicular to the beam propagation direction (*z*-axis). This is unlike traditional optical tweezers that are fundamentally point-traps and can trap one particle at a time. Several spherical beads undergoing Brownian motion in the solution are trapped by the lightsheet gradient potential, and the time (to reach trap-centre) is estimated from the video captured at 230 frames/s. High-speed imaging of beads with increasing laser power shows a steady increase in trap stiffness with a maximum of 0.00118 pN/nm at 52.5 mW. This is order less than the traditional point-traps, and hence may be suitable for applications requiring delicate optical forces. On the brighter side, light sheet tweezer (LOT) can simultaneously trap multiple objects with the distinct ability to manipulate them in the transverse (*xy*) plane via translation and rotation. However, the trapped beads displayed free movement along the light-sheet axis (*x*-axis), exhibiting a single degree of freedom. Furthermore, the tweezer is used to trap and pattern live HeLa cells in various shapes and structures. Subsequently, the cells were cultured for a prolonged period of time (> 18 h), and cell viability was ascertained. We anticipate that LOT can be used to study constrained dynamics of microscopic particles and help understand the patterned cell growth that has implications in optical imaging, microscopy, and cell biology.

## Introduction

Optical tweezers (OTs) are known to play critical roles in understanding molecular forces (or torques) and mechanical properties of proteins (DNA, RNA, and Lysozyme). In addition, OT has applications in optical sorting (sorting cells^1,2^, sorting colloidal spheres^3^), and single-molecule biophysics^4,5^. The delicate force exerted by radiation has consequences in precision measurement, determination of intermolecular forces, and short-distance interactions in the sub-piconewton range.

Arthur Askhin was the first to show that micron-sized latex spheres suspended in water can be manipulated using optical forces^6^. Subsequently, the first demonstration of single-beam optical tweezers was carried out, and successful trapping of bacteria and red blood cells was realized^7,8^. The basic physics of optical tweezers revolves around the fact that light carries linear and angular momentum, and this can be harvested to manipulate microscopic particles both inside and outside live cells. The forces that compete and need to be balanced for a stable trap are gradient and scattering forces. For his pioneering work on optical tweezers, A. Askhin received nobel prize in the 2018^9^. In recent years, many variants of optical tweezers are reported. Notably, line optical tweezers are reported and used successfully to observe liposome state transitions, cellular-liposome interactions, rotation of microscopic objects, and short-range colloidal interactions^10–13^. Off-late, specialized beams (Hermite-Gaussian, Bessel and Bessel–Gauss) are also theoretically proposed for trapping^14–17^. Previously, line traps are realized by a variety of optical means such as, scanning point-like traps^18^, holography^19,20^, or beam-shaping^12^. Although these technologies advanced the field, they need precise alignment, are inherently slow (due to the need for scanning), and are complex. With these constraints in mind, we developed LOT that is tunable, easy to align, and has new capabilities. Specifically, LOT can be used to optically trap a variety of objects (both spherical and elongated). For example, such traps are suitable for trapping live cells and model organisms such as *C. elegans*. Another advantage of LOT is its ability to simultaneously trap several particles or cells, and manipulate them (via translation and rotation) in the transverse plane. Overall, LOT is a new kind of optical tweezer primarily based on diffraction-limited light sheet. LOT can be further advanced, automated, and progressed into a cell-printing device.

Historically, light sheet was first generated by Siedentopf and Zsigmondy in the year 1903 to observe gold particles^21^. Later on, a modernized version of light sheet microscopy was built using cylindrical lens in 1993 by Voie et al.^22^. Subsequently, light sheet was diversified by Stelzer and Keller for biological imaging^23–26^. The technique has seen applications in diverse fields ranging from biological sciences (biological imaging^27–32^, iLIFE imaging cytometry^33,34^) to physical sciences (nanolithography^35,36^, optics^37,38^). The last decade has seen an explosion of light sheet variants that can be adapted for applications requiring optical manipulation. Some of these include thin light-sheet microscopy^39^, ultramicroscopy^40^, objective coupled planar illumination microscopy (OCPI)^41^, confocal light-sheet microscopy^42,43^, multiple light-sheet microscopy^44^, dual-inverted selective-plane illumination microscopy (diSPIM)^45^, light-sheet theta microscopy (LSTM)^46^, open-top light-sheet (OTLS)^47,48^ and lattice light sheet microscopy^49^, LVLSM^50^ and IML-SPIM^29^. These variants offer many useful configuration for generating light sheets that may be suitable for realizing application-specific optical manipulation system.

In this article, we propose and develop lightsheet optical tweezers (LOT) for trapping microscopic objects in a line. This is accomplished by generating a diffraction-limited light sheet using a combination of a cylindrical lens and a high NA objective lens. In traditional point-based traps, light is focused by spherical optics (such as high NA objectives) that produce point focus with maximum intensity at the center giving rise to a well-defined point trap. Both sub-micron and micron-sized particles can be trapped and manipulated using point traps. This is different for a cylindrical lens system that focuses light on a line rather than a point. As a result, the particle trapped in line-focus has a single degree of freedom. Using a combination of cylindrical and high numerical aperture objective lens, a stable diffraction-limited optical trap is realized, and the same is used for manipulating live HeLa cells.

## Results

### Lightsheet optical tweezer (LOT) system

**Figure 1:**
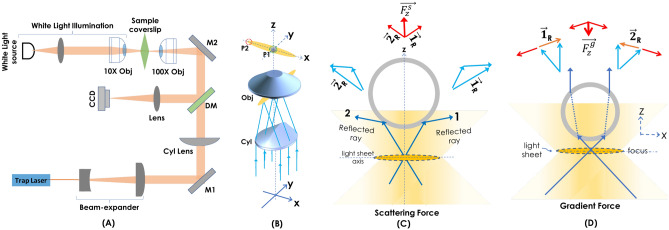
(**A**) Schematic diagram of the developed lightsheet optical tweezer (LOT) system. (**B**) The combination of key optical components (cylindrical lens and high NA objective lens) for generating diffraction-limited light sheet. (**C**) The resultant scattering force (due to the reflection of light) acts on the micro-particle along $+z$-axis. (**D**) The gradient force (due to refraction) acts inwards (towards the lightsheet axis) on the particle, i.e., along the $-z$-axis. The force diagrams shown with blue and red arrows indicate elemental and resultant forces, respectively. The picture of the actual optical trap is shown in Fig. S1 (see, Supplementary 1).

The schematic diagram of the developed optical tweezer (LOT) is shown in Fig. 1A trap laser of wavelength 1064 nm is used to trap dielectric silica beads (Thorlabs, USA). The laser beam is expanded 3 times by the beam-expander so as to fill the back-aperture of the cylindrical lens (Cyl Lens, $f=150$ mm). The lens focus light along *y*-axis on to the back-aperture of high NA objective lens (Olympus, 100 $X$, 1.25 NA). This results in the formation of diffraction-limited line-focus. A separate illumination sub-system is integrated for visualizing the specimen (beads and cells in solution). The illuminator consists of a white light source, a condenser lens, and a low NA objective lens (Olympus, 10 $X$, 0.25 NA). The lens illuminates a larger field-of-view (FOV) of the sample plane, and the transmitted light is collected by the 100 $\times$ objective lens. The light then pass through the dichroic mirror (DM) to the tube lens, which focuses it on the fast CMOS camera (Gazelle, Pointgray, USA). The actual LOT optical system is shown and discussed in Supplementary 1. A schematic of the key optical elements used in LOT for generating light sheet trap is shown in Fig. 1B. Note that the sheet formed by the cylindrical lens is projected on to the back-aperture of a high NA objective lens that gives rise to a diffraction-limited light sheet at the focus. The line focus is formed along the x-axis, orthogonal to propagating direction (along *z*-axis). The resultant field and trap geometry is shown in Fig. 1. Two major forces (gradient force and scattering force) act on a spherical bead, as shown in Fig. 1C,D. The scattering force ($\vec {F}_z^s$) on the particle is towards the beam-propagation direction (z-axis outwards) that pushes the particle away from the light sheet center (see, red arrow). The corresponding vector force diagram is shown in Fig. 1C. On the other hand, the gradient force is primarily due to refraction and exerts a restoring force on the particle when it is away from the center (lightsheet axis). So, the gradient force ($\vec {F}_z^g$) pushes the particle towards high intensity, which is the center of trap (see, red arrow). This is explained based on the vector force diagram shown in Fig. 1D. Unlike point-traps where particle experience gradient forces radially inwards (along $\vec {r}$), LOT involves gradient force along *y* and *z* directions only. This allows the particle to move freely along *x*-axis.

**Figure 2:**
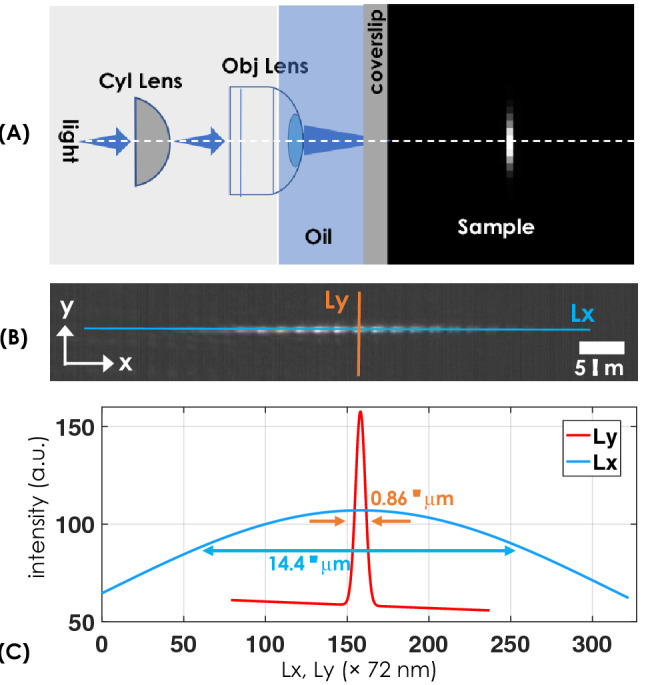
(**A**) Schematic diagram showing the formation of lightsheet point spread function (PSF) inside the specimen. This is realized by a combination of cylindrical lens and the oil immersion high NA objective lens. (**B**) The reflected image of lightsheet formed in the cell medium (cell medium) as observed by the camera. (**C**) Gaussian fit for the intensity across the light sheet (blue and orange line), shows the actual size of lightsheet to be $0.86 ~\upmu {\mathrm{m}} ~\times ~14.4 \upmu {\mathrm{m}}$ ($y\times x$).

### Light sheet point spread function (PSF)

Trapping micro-particles require a strong, stable, and confined optical field (system PSF). This necessitates high intensity light generated by a high NA objective lens. The schematic diagram of the key optical component, along with the formation of light sheet, is shown in Fig. 2A. The field is shown at discrete values of *z* in the specimen. Figure 2B displays the actual field recorded by the camera (in the reflected mode) for a combination of the cylindrical lens ($f=150$ mm) and high NA objective lens (100 $X$, 1.25 NA). Visually, the field displays optical aberration in the specimen medium, which is predominantly due to multiple reflections and medium inhomogeneity. Alongside, the intensity plots are also shown (see Fig. 2C). A Gaussian function is fit to the data to determine the dimension of the light sheet. The size of the light sheet is estimated to be $14.4 ~\upmu$m (FWHM) along *x*-axis and has a thickness of $0.86 ~\upmu$m (FWHM) along *y*-axis.

### Trap stiffness

**Figure 3:**
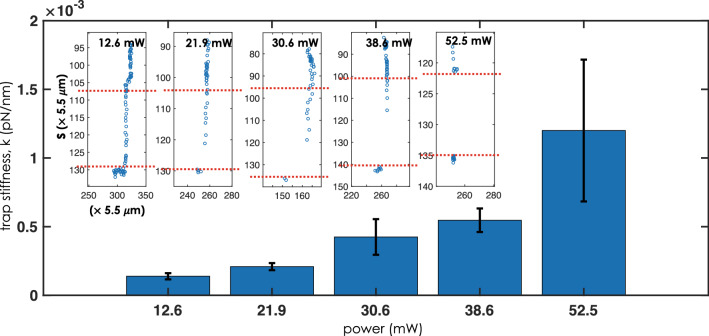
Trap stiffness *k* (pN/nm) of LOT at varying light intensity, 12.6–52.5 mW. The insets show the time taken by a single bead to reach the trap-center with increasing intensity, where the number of blue dots indicates the number of frames taken during the trapping process.

Experimental determination of trap stiffness begins with the initial condition that the beads are free in the medium and exhibit Brownian motion. In the presence of light sheet (at t = 0), some of the randomly moving beads sense the gradient potential and are directed towards the focus. The entire journey of the bead from time t = 0 (exhibiting free Browning motion) to the trap-center occurs fast, which is recorded by the high-speed CMOS camera (Gazelle, Pointgray, USA). Subsequently, the travel time (*t*) can be calculated from the number of frames (of the recorded video) between the initial position (t = 0) to the final position (trap-center). From the video, several free beads are marked and are tracked on their way to the trap-center to calculate the time (see, top and bottom dotted red line in Fig. 3). Dielectric beads take a large time or equivalently more number of frames (represented by blue dots) to reach trap-center at low light intensity (see inset in Fig. 3). This is understandable since low power produces a weak optical trap. Other important parameters include mass ($m= \rho V$) of the bead that can be calculated from the density of bead $\sim$ 2000 kg/m$^3$, and its volume (assuming spherical shape, $V=(4/3) \pi (d/2)^3$, where *d* is the diameter). The average time between two consecutive positions (*s*) of the bead in Fig. 3 or equivalently the between two frames (represented by blue dots in the track-plot) is, 9.4 ms. Knowing that, the viscosity of deionized water at $25^{\circ }$C is, $\eta \approx 0.8925 \times 10^{-3}$ Pa s, the average trap stiffness of LOT can be calculated using, $k= 6\pi \eta r_b v /t = 16.82 \times 10^{-9}$/t pN/nm. See, “methods section” for trap stiffness calculation. A better estimate can be arrived at by taking into account other forces related to internal flow and temperature. Figure 3 shows the trap stiffness (*k*) at varying light intensity, with a maximum of 0.00118 pN/nm at 52.5 mW. It is immediately evident that lightsheet traps are an order weaker than typical point traps^51,52^. This is predominantly due to the fact that light sheet point spread function (PSF) is spread over a larger space (here along a line) when compared to point-PSF employed in traditional tweezer, and so the intensity is much weaker than that of a typical point-trap for the same power.

### Trapping dielectric beads

**Figure 4:**
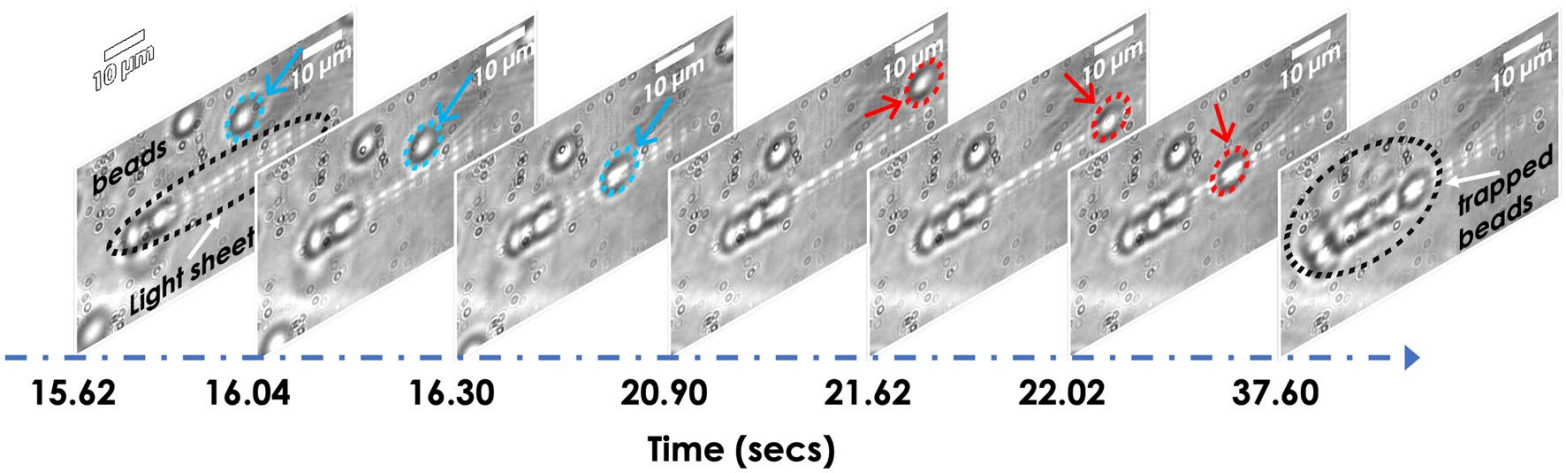
Images taken during the trapping of dielectric beads. Few free beads in close proximity to the light sheet are indicated by blue and red circle/arrow that are eventually trapped in the process. The respective timeline is shown below. The entire process is encapsulated in the Supplementary Video 1.

To visualize the functioning of LOT system, we used silica beads suspended in deionized water as a sample. The bead solution is dropped on the glass-coverslip, and then it is placed on the oil-dipped 100 $X$ objective lens. The light sheet is generated in the solution, as shown in Fig. 4. The beads can be seen randomly distributed, with two beads lying on the light sheet. At the time $t=15.62$ s, few beads are seen trapped, and one free bead (marked by blue arrow and circle) is in the proximity of light sheet. In the frame (recorded at 16.30 s), the bead is seen trapped by the gradient potential. The next frame (taken at $t=20.90$ s) shows an approaching free bead (marked by the red arrow and circle) which is eventually attracted by the potential in frames (taken at 21.62 s). Subsequently, the bead slide down to the other end of light sheet (frame at 22.02 s) due to slight tilt in the sample holder. Over time a number of beads are arranged on a line (light sheet), as seen from the image taken at 37.60 s. The entire trapping process can be visualized in the Supplementary Video 1. On an average, dielectric beads took 0.70 s to reach the trap center (axis of lightsheet PSF).

**Figure 5:**
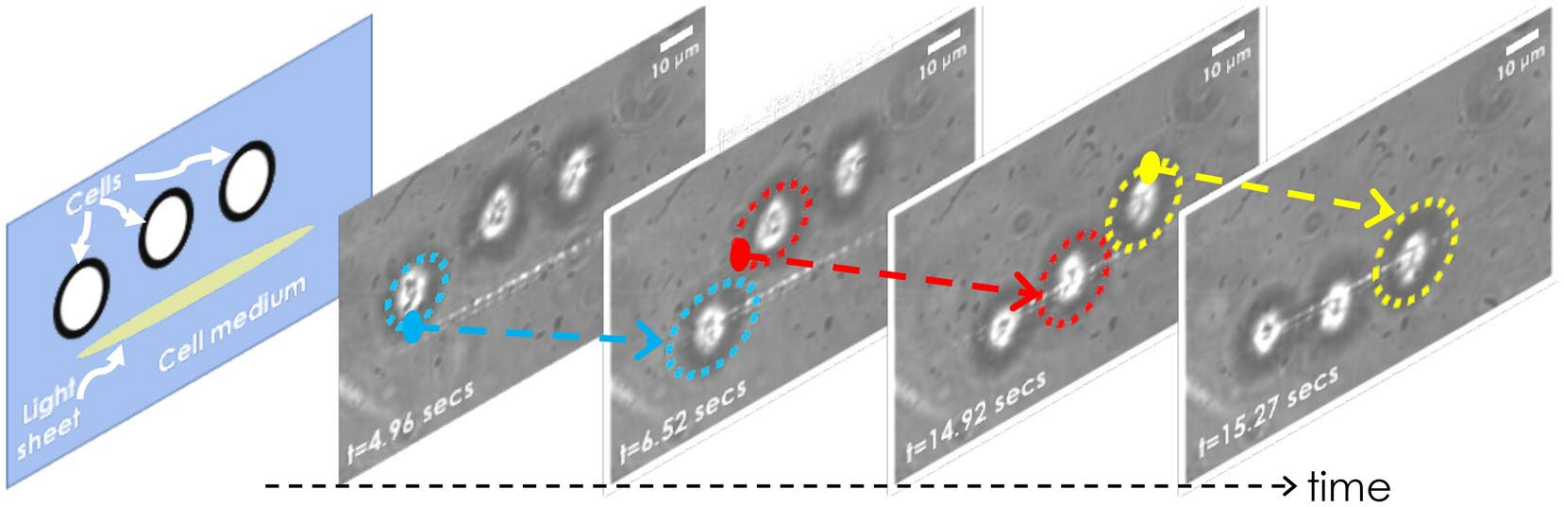
LOT for trapping freely moving HeLa cells suspended in cell medium (DMEM). The randomly moving cells were trapped one-by-one and aligned in a line. See, Supplementary Video 2.

### Trapping live HeLa cells

Similar to dielectric beads, live HeLa cells were trapped. Live cells were suspended in the cell medium and thoroughly pipetted. A small amount (about $10~\upmu$l) is dropped on the coverslip, which is attached to the 3-axis nanopositioning stage (MAX3SLH, Thorlabs, USA). Subsequently, light sheet is generated near the coverslip surface for trapping, patterning and culturing live cells. The cells in the proximity were attracted by the gradient potential of the lightsheet field. The corresponding video of cell trapping is shown in Supplementary Videos 2 and 3. The HeLa cells are heavier than the beads, so they are relatively slow to move and take more time to reach the trap center (attractive potential). Experiments show that the following relation holds good: $m / t = p_0$, where *m* is the mass of the object, *t* is the time it took to reach the trap center, and $p_0$ is a constant related to the trap-stiffness. This states that a large mass takes more time to reach the trap center and vice-versa. At the time $t=0$, the cells were freely moving in the medium (see, Fig. 5) but started to move towards the light sheet as the laser was switched on. Using a high-speed camera (Gazelle, Pointgray, USA) operating at 10 ms/frame, HeLa cells took an average of 8.57 s time to reach the trap center. In comparison to the dielectric bead (with an average of 0.7 s), Hela cells took an average of 8.575 s to reach the trap center. This is approximately 12.25 times slower than dielectric beads.

### Live HeLa cell patterning and patterned cell growth

**Figure 6:**
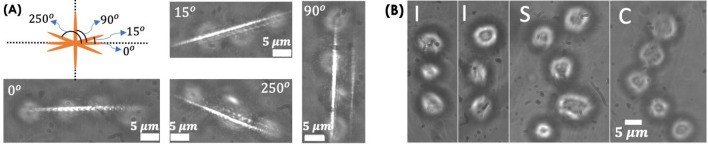
(**A**) Oriented light sheet for patterning at any desired slope. Specifically, trapping and patterning of cells at $0^{\circ }$, $15^{\circ }$, $90^{\circ }$ and $250^{\circ }$ in the transverse plane are shown. (**B**) Writing the pattern “IISc” using LOT.

**Figure 7:**
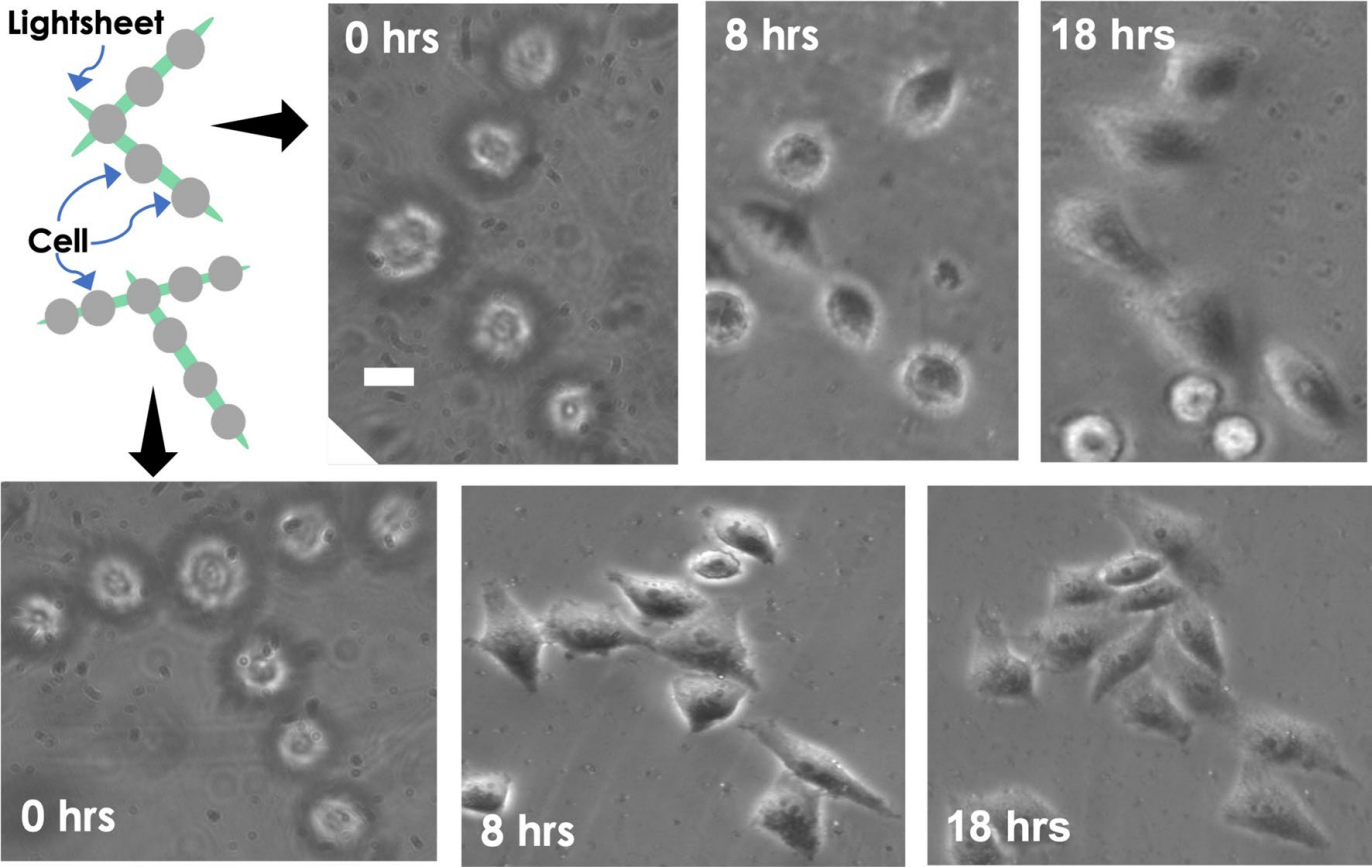
HeLa cells patterned in specific shapes (L and T). Subsequently, the patterned cells were cultured for a longer duration (8 h and 18 h) in a standard cell culture incubator maintained at $37^{\circ }$C and 5% CO$_2$. The scale is 5 µm.

Patterned cell growth plays a critical role during the early development of multicellular organisms. This is essential for cells to communicate with each other that control its growth at a healthy rate. Uncontrolled growth is known to occur in cancer. In the present study, we have considered HeLa cancer cells. The cells were thawn and grown in a 35 mm disc supplemented with cell medium (DMEM + FBS). To detach them from the surface, the cells were tripsinated, followed by centrifugation and resuspension according to standard sample preparation protocols^31^. Subsequently, the floating live cells (spherical shape) were subjected to lightsheet trap. One-by-one the cells were trapped by the light sheet field and aligned in a line as displayed in Fig. 5 (see blue, red, and yellow arrow). The corresponding timeline is also indicated, and the entire trapping process can be visualized in the Supplementary Video 2. The results show that the technique can pattern cells in a preferential direction (along a line). In addition, light sheet can be rotated in the transverse plane, facilitating patterning at any desired angle. Figure 6A displays the cells at $0^{\circ }$, $15^{\circ }$, $90^{\circ }$ and $250^{\circ }$. This is a prerequisite for patterning complex structures such as writing “IISc” as shown in Fig. 6B. Furthermore, the technique allows patterned growth of cells in specific shapes (T and L) and enables sustained culturing for long hours (up to 18 h), as shown in Fig. 7. Although we see outgrowth and deposition of some random cells (floating in the medium) on the patterned cells, the cells were found to be healthy for > 18 h. This shows that LOT is a promising technique for cell trapping, patterning, and culturing, all on a single platform.

## Discussion

A lightsheet based optical tweezer is proposed and demonstrated for the first time. Unlike existing optical traps, LOT uses light sheet as a PSF to trap objects. The technique is shown to trap dielectric beads and live HeLa cells without causing any adverse effect on cell viability. The ability of LOT to trap several objects simultaneously expands its use beyond traditional point-based optical tweezers. In fact, the technique has facilitated patterning of live cells in various shapes, thereby in-principle can mimic tissue formation from the basic unit of life (cell).

Unlike traditional point-trap tweezers, LOT has the ability to trap multiple objects in a line. While this accelerates the trapping, it must be realized that the trap is an order weaker than the traditional optical traps. This is predominantly due to the redistribution of intensity in a sheet compared to a point. On the other hand, LOT has the advantage of accessing weak forces, and simultaneously trap multiple objects. But the technique require an order more laser power. Our observation show that LOT requires a power of 52 mW (at the objective) as compared to < 5 mW that is generally used in traditional traps^53,54^. In addition, the technique requires a combination of cylindrical lens and high NA objective lens for generating diffraction-limited light sheet that forms a stable and intense light sheet PSF (see, Figs. 1 and 2).

To demonstrate the capability of LOT for optical trapping, we used dielectric beads suspended in distilled water and HeLa cells in a medium. While beads are found to trap faster, the cells took more time to reach the trap-center, which is due to its large mass (see, Supplementary Videos 1 and 2). Another practical issue associated with multiple cell trapping is their tendency to attach just before they come under the influence of gradient potential, thereby making it hard to trap them individually (see, Fig. 5). It may be noted that we did not use Tripsine in the cell medium to avoid clumping. This ensures the viability of cells for prolonged biological studies related to transfection and drug treatment. Moreover, this facilitates the analysis of cells in their natural environment (cell medium, 5%  CO$_2$, and $37^{\circ }$C).

The foremost step to cell patterning is to trap multiple cells in the light sheet PSF and the ability to manipulate (translate and rotate). While translation is easy to achieve, the rotation of cells (trapped in light sheet) is accomplished by precisely turning the cylindrical lens in the illumination sub-system. We demonstrate the trapping of beads and HeLa cells in a lightsheet and its rotation in the transverse plane. Both translation and rotation form a preamble to pattern cells in specific shapes. Moreover, LOT is successfully used to write alphabets and even words using cell (see, Fig. 6). Apart from trapping and patterning, LOT facilitates prolonged cell culture and sustained growth over a long period of time (18 h and beyond). This is demonstrated by patterning HeLa cells in specific shapes and culturing them for up to 18 h (see, Fig. 7, and Supplementary 2). Moreover, the cells were found to be healthy for carrying out biological studies even beyond this period. This suggests that large-scale trapping and patterning can be achieved efficiently using LOT compared to a traditional point-based optical tweezer.

LOT may be helpful in applications requiring directional and patterned cell growth. Specifically, the technique can be used to understand the continual unregulated proliferation of cancer cells^55,56^. Cancer cells predominantly grow and divide in an uncontrolled manner in all directions, whereas normal cells respond appropriately to the signals that control directional cell growth. In this respect, patterned cell growth studies may help understand this behavior. Another application where patterned cell growth can be of potential use is a neural network^57^ and brain-on-chip model^58^. The recreation of neural networks with designed topology has proven to be a valuable tool to decipher the behavior of neurons in a hierarchical network. Patterning could help understand this behavior at a few neurons level to an entire complex neural network and may prove to be paramount for studying brain activity. In addition, the technique may be helpful for applications requiring directional cell growth^59^ and wound healing^60^.

With the ability to simultaneously trap several particles (both living and non-living), and scalability, LOT is expected to advance the field of optical manipulation. Moreover, the LOT-PSF makes it suitable to trap both cells and elongated objects such as *C. elegans*. This expands the horizon of LOT for applications that necessitates study on free live organisms without the need for immobilization using glue or anesthetics^61,62^. LOT is expected to accelerate the field of optical manipulation, biophysics, and cell biology.

LOT is a new kind of optical tweezer primarily based on light sheet geometry. The technique is faster, stable, and capable of capturing multiple particles in a line/plane when compared to traditional optical tweezers. To the best of our knowledge, this is the first light sheet based tweezer system and has never been reported before for optically trapping applications. The technique is expected to further optical manipulation. It may enable new applications in diverse disciplines of physical (atomic and colloidal physics) and biological sciences (single-molecule biophysics and organism biology). In the future, the technique may be optically modified to realize planar traps where particles can be trapped in an entire plane (generated by the sheet of light).

## Methods

### The theory of light sheet optical trap

The theory of LOT is similar to that of a constrained motion along two axes (propagation direction and perpendicular to light-sheet axis) and free movement along the light sheet axis. Unlike point-traps that are better understood in the cylindrical coordinate system (*r*,  *z*, with *r* being the lateral/radial plane and *z* being the beam propagation direction), LOT is better understood in a rectangular coordinate system (*x*,  *y*,  *z*) as shown in Fig. 1. The diffraction-limited light sheet is shown along *xy*-plane with *x* and *z* as the lightsheet axis and beam propagation direction, respectively. In Fig. 1, the cylindrical lens focus light on a line extending along *y*-axis (see, yellow oval just before the back-aperture of the objective lens in Fig. 1B). The objective lens is placed at the focus of a cylindrical lens. The field at the back-aperture of the objective lens undergoes Fourier transform, forming a diffraction-limited line at its focus (see, yellow oval along *x*-axis at the focus of the objective lens in Fig. 1B). In general, two cases arise: (1) the particle is much smaller than the wavelength of light (Rayleigh regime), and (2) the particle is larger than the wavelength of light (Geometric regime). In the Rayleigh regime, the corresponding force along *y* is given by, $\vec {F}_y^g = \frac{\partial U}{\partial y}= -\alpha \frac{ \partial }{\partial y} I(x,y,t) =-\alpha \frac{ \partial }{\partial y} \langle \vec {E}(y,t)^2 \rangle = -\frac{\alpha }{2} \frac{\partial }{\partial y} |E_0(y,t)|^2 = \beta \frac{\partial }{\partial y} I_0 (y,t)$, where, $\langle \cdots \rangle$ is the time average, and $\langle \vec {E}(x,y,t) \rangle = |E_0|^ /2$. Note that the variation of intensity along *x* is negligible and does not change appreciably, except at the far ends. Hence, $\vec {F}_x = \frac{\partial U}{\partial x} = \beta \frac{\partial }{\partial x} I_0 (x,t) = 0$. In the above expression, we have absorbed the refractive index and permittivity in a single parameter, $\beta =\alpha / 2cn \epsilon _0$, where *n* is the refractive index of the particle and $\epsilon _0$ is the permittivity of vacuum. Accordingly, the particle is trapped when the polarizability of the particle is greater than the surrounding media. In the geometric regime, where the particle size is larger than the wavelength of light such as dielectric beads, ray-optics can be employed to understand forces acting on the particle. Classically, force on the particle can be defined as the rate of change of momentum, $F= \frac{\partial \vec {p}}{\partial t}$, where $\vec {p}$ is the momentum of the particle. The conservation of momentum necessitates the exchange of momentum between light and the particle. However, the off-focal beads experience a net force towards the trap-center (high-intensity region) due to gradient force, as explained by the force diagram in Fig. 1D. A similar force but in the opposite direction appears when the particle is on the other side of light sheet axis. Next, let us determine the role of scattering force $F_s$. Primarily, the scattering occurs due to reflection of light, and the scattering force in Rayleigh regime can be expressed as, $\vec {F}_s = \frac{n_m ~\sigma _s}{c} ~\langle \vec {S}_i \rangle$, where, $\sigma _s$ is the cross-section of particle. This means that scattering forces are directly proportional to the cross-section of particle. So, large particles experience a greater scattering force. Figure 1C shows elemental forces and the resultant scattering force ($\vec {F}_z^s$) due to the reflection of light at the bead surface. This points in the direction of Poynting vector $\vec {S}_i$ which is also the direction of beam propagation (z) and has a unit of energy per unit area per unit time. So, the scattering force has the direction of Poynting vector. Accordingly, scattering (due to reflected light at the bead surface) results in momentum transfer between light and particle that tends to push the particle out of focus with force, $\vec {F}_z^s$ (see, Fig. 1C). The gradient force with its maximum at the trap-center leads to a stable trap along the beam propagation direction (*z*-axis). A similar explanation is true for *y*-axis as well. Overall, the gradient forces are along $y-z$; however, the bead is free to move along *x*-axis due to negligible intensity gradient. Thus, the condition for a three-dimensional stable trap along the line-focus is realized when the gradient potential overcomes the other forces (radiation pressure or scattering force, buoyant force, and the forces due to Brownian motion and gravity).

### Calculation of trap stiffness

We used spherical silica beads (size $\sim 2 ~\upmu$m) suspended in deionized water as the sample to estimate trap stiffness. In general, suspended particles/beads undergo random Brownian motion but follow a directed motion (towards the trap center) under the influence of gradient potential. To a good approximation, an optical trap behaves like a harmonic potential, and it is able to exert a restoring force. Specifically, near the trap center, the force can be approximately modeled by Hooke’s law, and the restoring/gradient force is given by $F(x) = -kx$, where *k* is the trap stiffness (N/m) and *x* is the displacement from trap-center. The second force acting on the particle is viscous drag force. Assuming spherical beads, the particle moving through the fluid experiences a viscous force of, $F_{vis} = -6\pi \eta r_b v$. For simplicity and calculating approximate trap-stiffness, we neglect the effect of gravity on the bead, so we can ignore forces due to weight and buoyancy. Thus, the motion of the bead is governed by these two forces (gradient and viscous/drag force), which are opposite. Balancing optical (gradient) forces with drag force produces^63,64^, $-kx = -6\pi \eta r_b v \Rightarrow k= 6 \pi \eta r_b (v/x) ~~\Rightarrow k=6\pi \eta r_b /t$, where $x=vt$ and, *v* is the dragging velocity, *x* is the displacement and *t* is the time, $\eta$ is the medium viscosity, and $r_b = d/2$ is the bead radius. Given the dynamic condition (internal flow and cell dynamics) of the study, the proposed technique has given us a good estimate^63,64^. Here, we use this relation to determine the trap stiffness.

### Sample preparation

#### Dielectric beads

We have purchased non-Functionalized Fused Silica Beads in Deionized Water from Thorlabs, USA. Subsequently, the beads (of size, $2.06\, \upmu$m) are diluted in distilled water to one-fourth of the original concentration (2 g/ml) for trapping experiment.

#### Cell line and maintenance

HeLa cells (human cervical carcinoma cell line) obtained from our collaborator Dr. Upendra Nongthomba (Biological Sciences, Indian Institute of Science, Bangalore, India) were used for the experiment. The HeLa cells were cultured and maintained in incubator in complete Dulbecco’s modified minimal Eagle’s medium (DMEM) (Gibco, Thermo Fisher Scientific) supplemented with $10\%$ FBS( Gibco, Thermo Fisher Scientific) and $1\%$ penicillin–streptomycin solution (Gibco, Thermo Fisher Scientific) at $37^{\circ }C$ and 5%  CO$_2$ (CO$_2$-incubator, Thermo Scientific). Hemocytometer is used to count cells after every passage and approximately 100,000 cell count was maintained. The cells were passaged in every 2–3 days to maintain healthy cell lines. After two passages, the cells were tripsinated using 3.7% Tripsine which is followed by 4 min incubation. The cells were then pipetted to break-down cell-clusters followed by centrifugation at 3000 rpm. The supernatant is then pipetted out, and the cell-pallet were resuspended in cell medium. These cells were kept for 15 minutes in the incubator before carrying out trapping experiments.

## Supplementary Information

Supplementary Information.

Supplementary Video 1.

Supplementary Video 2.

Supplementary Video 3.

## Data Availability

All data generated or analysed during this study are included in this published article [and its supplementary information files].
