# Supplementary material for: Lightsheet optical tweezer (LOT) for optical manipulation of microscopic particles and live cells
Source: Sci Rep. 2022 Jun 17;12:10229. doi: 10.1038/s41598-022-13095-3 (PMC9205896; doi:10.1038/s41598-022-13095-3)
Supplement: Supplementary file 1 — Supplementary Information. [file 41598_2022_13095_MOESM1_ESM.pdf]

Title: Lightsheet Optical Tweezer (LOT) for Optical Manipulation of Microscopic Particles and Live Cells

Authors: Partha P. Mondal, Neptune Baro, Ankur Singh, Prakash Joshi and Jigmi Basumatary

Department of Instrumentation and Applied Physics, Indian Institute of Science, Bangalore 560012, INDIA

Supplementary Material

Supplementary 1: Actual Optical Setup of Lightsheet Optical Tweezer

Supplementary 2: Patterning and Culture of Live HeLa Cells Using LOT

Supplementary Videos

Supplementary Video 1: Trapping dielectric beads using LOT

Supplementary Video 2: Trapping HeLa cells – Set 1

Supplementary Video 3: Trapping HeLa cells – Set 2

Supplementary 1 | **Actual Optical Setup of Lightsheet Optical Tweezer**

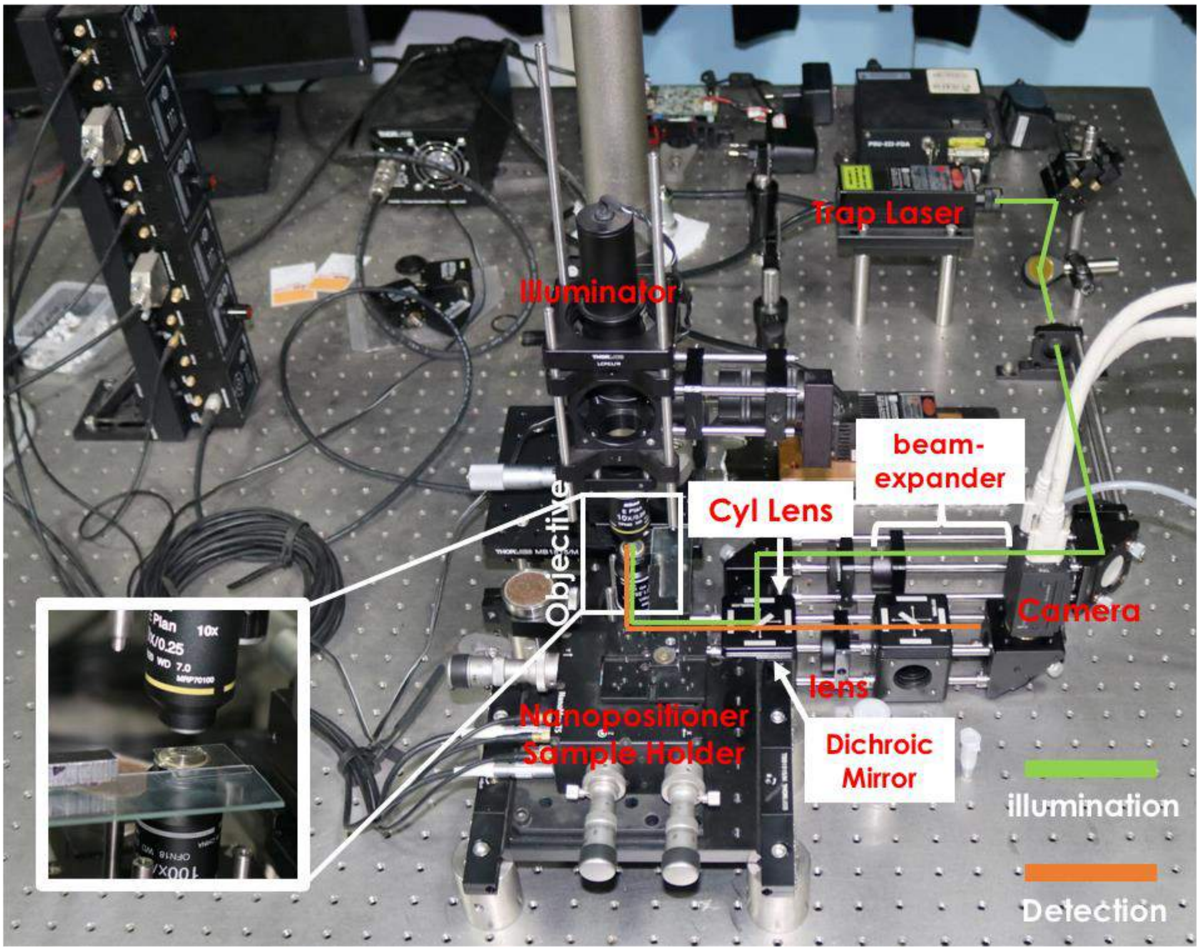

Fig. S1: The actual experimental setup of LOT system, where illumination and detection arms are indicated by green and orange lines, respectively. Most of the key optical components are also indicated.

The actual optical setup for lightsheet optical tweezer (LOT) is shown in Fig. S1. A high-power laser (maximum power 500 mW) with a trapping wavelength of 1064 nm is used to trap beads and live cells. The light is directed to the cylindrical lens through a series of IR mirrors (both adjustable and fixed mirror holders). The beam is aligned to the center of 25 mm optics using components purchased from Thorlabs. The cylindrical lens (of focal length, f=150 mm) is positioned to focus the beam at the back aperture of the objective lens. This results in the formation of diffraction-limited tightly-focused light-sheet at the working distance of the objective lens. We used a high NA objective lens for tight focusing of the beam. This results in a line focus rather than a point focus. The samples (beads and live Hela Cells) are dropped on

a coverslip attached to the glass slide, which is placed on a nano-positioner (Thorlabs, USA) for fine movement of the sample stage. A top white light illuminator is used to visualize the beads in the camera. On its way to the detection, the trap laser light is filtered out by the dichroic mirror. Due to less than 80% transmission efficiency of the filter, we can see part of the reflected light sheet as well. The dichroic mirror in combination with a short pass filter prevents backscattered light from the 1064 nm laser from saturating the CMOS sensor. Trapping of silica beads and HeLa cells are successfully achieved, as shown in Supplementary Video 1 and 2.

<u>Supplementary 2</u> | Patterning and Culture of Live HeLa Cells Using LOT

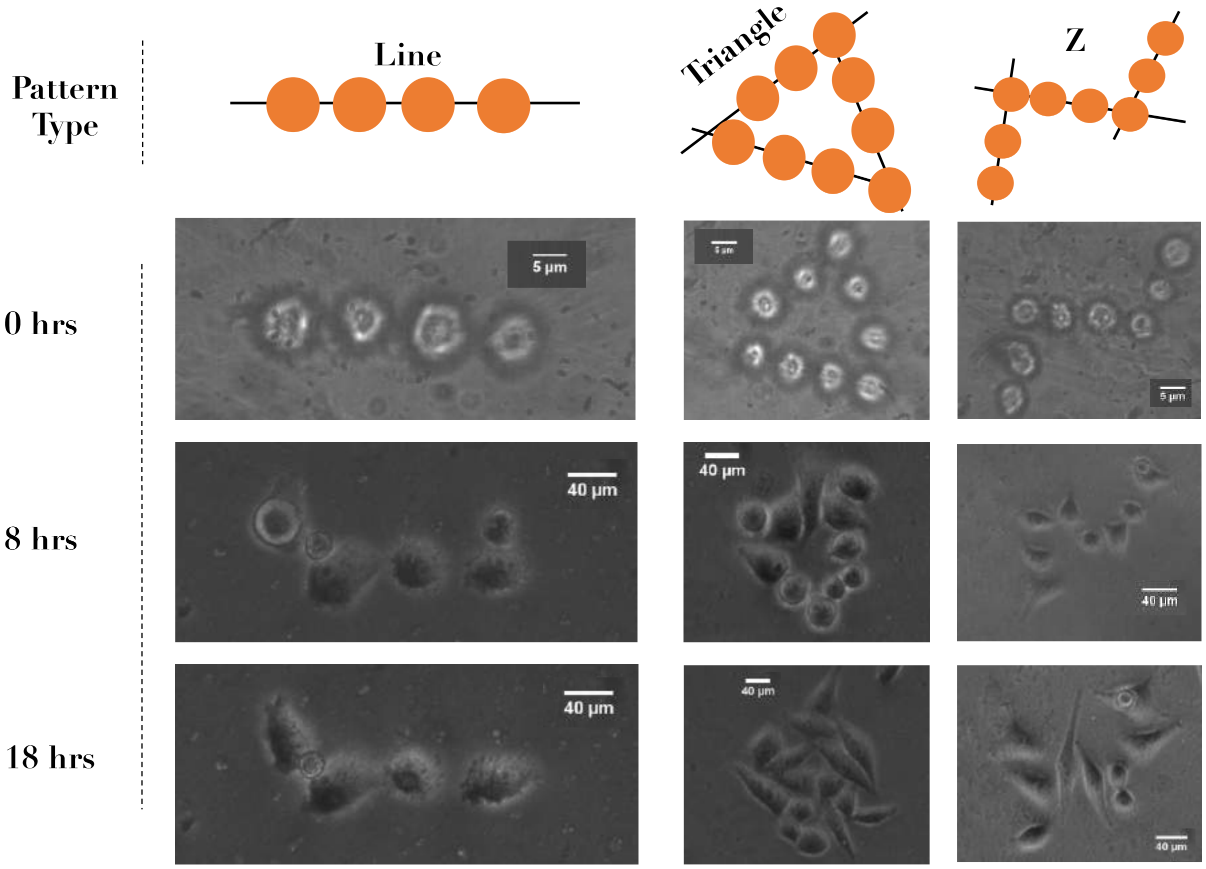

Fig. S2: LOT enables patterning of live HeLa cells in a line, triangle, and Z shapes. Post patterning, the cells were cultured for 8 hrs and 18 hrs.

Light-sheet enables rapid patterning of live cells in various configurations, as shown in Fig. S2. This has implications in a range of applications ranging from cell-cell communication, cancer biology, and neural networks [1][2][3][4][5]. Cells were prepared following the protocol described in the main manuscript (Methods section). The first step involves trapping Hela cells in a line by the light sheet PSF. Subsequently, the light sheet is translated and rotated (by precisely turning the cylindrical lens) to achieve desired position and angle. This is taken as the preamble to generate complex patterns such as triangle and Z. It is evident that, cells growth mostly happens inside the closed structure of the triangle, whereas for an open shape (such as "Z"), the growth mostly follows the pattern.

Overall, LOT enables controlled and organized growth of cells in user-defined patterns, and enables long time viability studies on these cells. We anticipate that further development of LOT will lead to cell printing applications.

Reference

1. H. Jin, J. Varner, Br J Cancer 90, 561–565 (2004). \bibitem{wirtz} D. Wirtz, K. Konstantopoulos, P. C. Searson, Nat Rev Cancer 11, 512–522 (2011).
2. D. Cabrera-Garcia, D. Warm, P. de la Fuente, M. T. Fernández-Sánchez, A. Novelli and J. M. Villanueva-Balsera, Sci, Rep. 11, 20407 (2021).
3. C. Forro, Davide Caron, Gian Nicola Angotzi, Vincenzo Gallo, Luca Berdondini, Francesca Santoro, Gemma Palazzolo, Gabriella Panuccio, Micromachines (Basel), 12, 124 (2021).
4. C. Kirchhelle, D. Garcia-Gonzalez, N. G Irani, A. Jerusalem, I. Moore, eLife 8, e47988 (2019).
5. P. Martin, Science 276, 75–81 (1997).
